# Volume-targeted on high-frequency oscillatory ventilation in preterm infants: a systematic review

**DOI:** 10.1016/j.jped.2025.01.012

**Published:** 2025-03-17

**Authors:** Eduardo Antonio de Sousa Orlandin, Thais Iwashita-Lages, Luis Kanhiti Oharomari-Junior, Milena Ramos Tomé, Mariana Tosato Zinher, Sofia Oliveira Dias, Walusa Assad Gonçalves-Ferri

**Affiliations:** a Hospital das Clínicas de Ribeirão Preto da Universidade de São Paulo, Departamento de Pediatria, Divisão de Terapia Intensiva Neonatal, Ribeirão Preto, SP, Brazil; b Universidade Federal de Campina Grande, Faculdade de Medicina de Campina Grande, Campina Grande, PB, Brazil; c Hospital Infantil Joana de Gusmão, Divisão de Oftalmologia, Florianópolis, SC, Brazil; d Faculdade de Ciências Médicas de São José dos Campos, Departamento de Medicina, São José dos Campos, SP, Brazil

**Keywords:** High-frequency ventilation, Volume-targeted, Premature infant, Respiratory distress syndrome, Lung protection

## Abstract

**Objective:**

This systematic review aimed to analyze, in neonates, the effects of high-frequency oscillatory ventilation (HFOV) with volume-targeted (VT) compared with conventional HFOV.

**Sources:**

The authors searched PubMed, EMBASE, Cochrane, and ClinicalTrials.gov from inception until August 4th, 2024, to identify studies comparing HFOV with and without VT in neonates under 44 weeks corrected age. Outcomes analyzed were VThf, amplitude and carbon dioxide partial pressure (PCO_2_) variability, episodes of hypoxemia, hypocarbia or hypercarbia, duration of mechanical ventilation, rates of bronchopulmonary dysplasia (BPD) or intraventricular hemorrhage (IVH), and mortality. ROB-2 and ROBINS were used for risk of bias assessment.

**Summary of the findings:**

This systematic review included 260 preterm infants from two crossover and four cohort studies. Five studies were considered as having a relevant risk of bias. Meta-analysis could not be performed, due to the differences in study design and incomplete reporting. The report of included studies indicates that HFOV with VT, compared with HFOV, may reduce VThf variability, hypocarbia and hypercarbia incidence. Findings on hypoxemia incidence and mechanical ventilation duration are mixed. Two studies found no difference in BPD rates, while one noted higher survival without BPD grades 2–3 under HFOV with VT. IVH, leukomalacia, and mortality outcomes were similar.

**Conclusions:**

Inclusion of VT during HFOV may reduce VThf variability, hypocarbia and hypercarbia incidence. However, there is a need for randomized trials to compare clinical outcomes from both ventilatory strategies.

## Introduction

In neonates, volutrauma is a leading cause of lung inflammation.^1^ Concerns about volutrauma triggered the development of new invasive mechanical ventilation strategies. These modalities include conventional ventilation with volume-targeted (VT), which delivers more precise tidal volumes, and high-frequency oscillatory ventilation (HFOV), which provides tidal volumes less than the dead space volume.^2^^,^^3^ Currently, both are recommended as first-line strategies for preterm infants with respiratory distress syndrome who require invasive ventilation.^4^

About conventional ventilation with VT, in addition to the lower volutrauma, the more precise tidal volume reduces the variation of carbon dioxide partial pressure (PCO_2_), consequently maintaining a more stable cerebral blood flow and reducing the risk of brain injuries. A meta-analysis found that infants ventilated using this modality, when compared to conventional pressure-limited ventilation, had lower rates of death, bronchopulmonary dysplasia (BPD), severe cranial ultrasound abnormalities, hypocarbia, and duration of ventilation.^5^

In the case of HFOV, the reduction in volutrauma is associated with the lower tidal volume provided.^6^^,^^7^ Compared to pressure-limited ventilation, randomized trials yielded lower pulmonary hemorrhage rates and duration of ventilatory support in the neonatal period and superior lung function in adolescence.^8^^,^^9^ However, a meta-analysis comparing these ventilation modalities found only a small reduction in the risk of BPD with HFOV, along with an increased risk of acute air leaks such as pneumothorax or interstitial emphysema.^10^

In HFOV, the high-frequency tidal volume (VThf) is directly proportional to the amplitude and inversely proportional to the applied frequency. Several factors can cause undesirable fluctuations in VThf. For example, changes in lung compliance at a fixed amplitude can alter the delivered VThf, while increased secretions can disrupt the oscillatory flow and hence reduce VThf. Additionally, spontaneous breathing during ventilation may result in fluctuations in VThf. These variations increase the risk of air leaks. Also, they can destabilize PCO_2_ levels, leading to dangerous variations in cerebral blood flow, and compromising neuroprotection.^7^

In this sense, a new strategy has been proposed to combine HFOV and VT, fixing VThf at a target value. This modality can deliver a more stable VThf, as the amplitude is continuously adjusted based on the last expiratory volume obtained. In a fixed VThf, PCO_2_ washout is directly proportional to the frequency, similar to conventional ventilation.^11^^,^^12^

Despite the proposal of HFOV with VT in reducing disturbances in VThf and CO_2_ removal, its benefits compared to conventional HFOV remain unclear in preterm infants [7,13]. Therefore, the authors performed a systematic review to assess if, in preterm infants under 44 weeks of corrected age, clinical and ventilatory outcomes differ between HFOV with and without VT.

## Sources

This systematic review was performed following the Preferred Reporting Items for Systematic Reviews and Meta-Analyses (PRISMA) statement guidelines and the Cochrane Handbook of Systematic Reviews of Interventions recommendations.^14^^,^^15^

The authors systematically searched PubMed, Embase, Cochrane Library, International Clinical Trials Registry Platform (ICTRP), and ClinicalTrials.gov to identify eligible studies. In addition, references from possible included publications were evaluated for additional studies. The search was delimited from inception until August 4th, 2024, in all the databases mentioned. The full search strategy applied was: (neonatal OR neonate OR neonat* OR newborn OR premature OR preterm OR infant OR infants) AND (high-frequency OR “high frequency” OR “high frequency ventilation” OR “high frequency oscillatory ventilation”) AND (“volume guarantee” OR “volume-guarantee” OR “volume targeted” OR ”volume-targeted”). No time limit was determined for the research process.

Two authors independently performed the study selection process, data extraction from eligible articles, and quality assessment. All conflicts were resolved through consensus. Inclusion criteria were randomized or non-randomized studies that compared HFOV with and without VT, in neonates under 44 weeks corrected postnatal age. Studies that evaluated only HFOV with VT or without VT were excluded (Supplementary Table).

Two authors independently performed the quality assessment, using the revised Cochrane risk-of-bias tool for randomized trials (ROB2), and the Risk of Bias In Non-Randomised Studies of Interventions (ROBINS-I) for randomized and observational studies, respectively.^16^^,^^17^ Disagreements were resolved through consensus.

Rates of BPD at 36 weeks postmenstrual age were defined as the primary outcome. Secondary outcomes were VThf, amplitude and carbon dioxide partial pressure (PCO_2_) variability, episodes of hypocarbia, hypercarbia or hypoxemia, duration of mechanical ventilation, rates of intraventricular hemorrhage (IVH), and mortality.

For included studies, the inclusion criteria were summarized, and baseline characteristics were manually collected with a Microsoft Excel® spreadsheet by two researchers. The main outcomes of each study were extracted and summarized. If three or more studies presented a low risk of bias, a meta-analysis would be performed.

## Results

**Figure 1:**
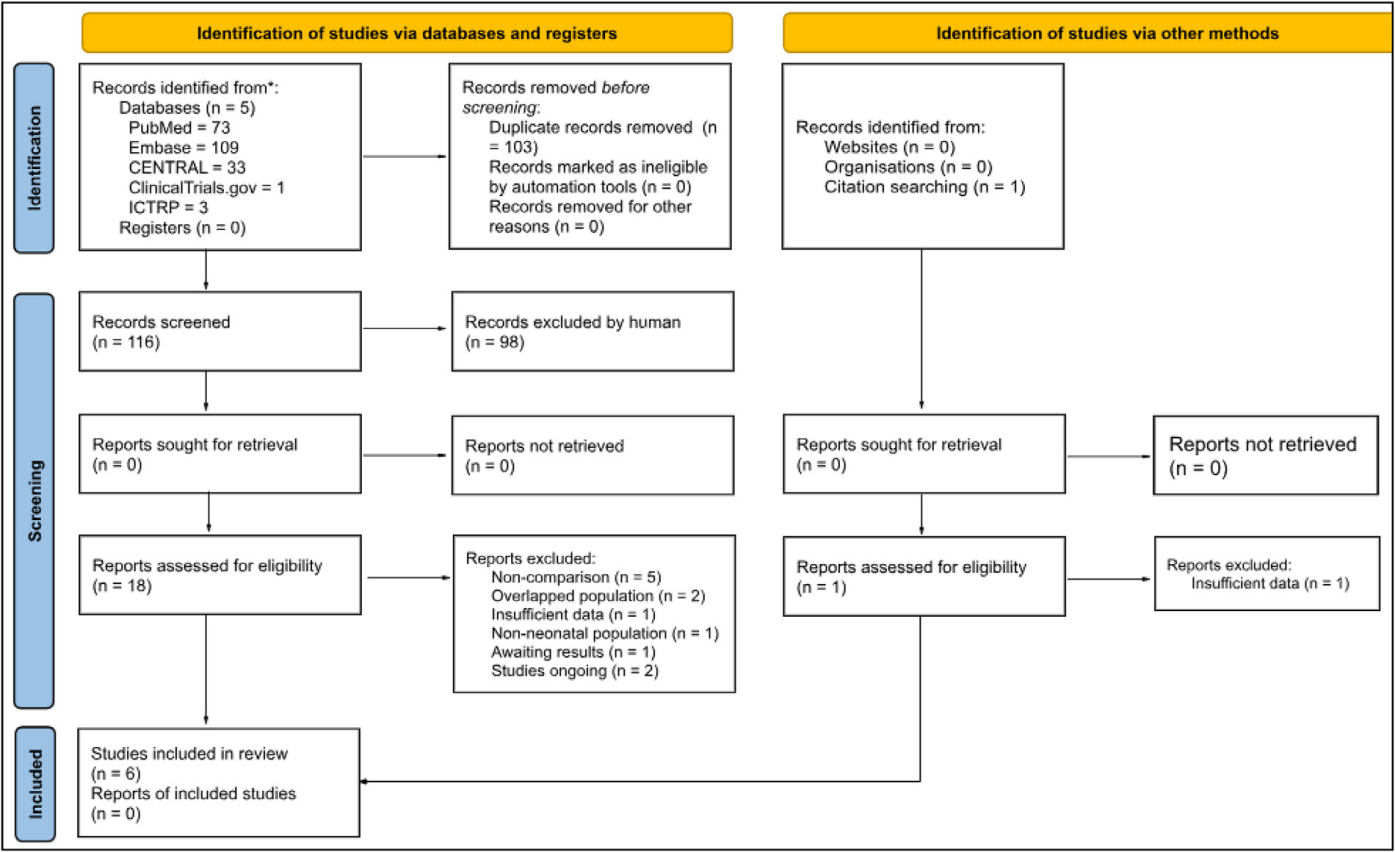
PRISMA 2020 flow diagram.

After removing duplicates, 116 articles were identified, and 18 studies were selected for full-text review after exclusion by analysis of title and abstract (Figure 1). One additional study was identified through a citation search from a potential study, but it was excluded due to insufficient data.^18^^,^^19^ Ultimately, six studies met the eligibility criteria, comprising 260 preterm infants. A summary of the included studies and the main findings are available in Table 1. Studies ongoing and awaiting assessment are available in Table 2.

**Table 1:** Summary of included studies.

**Study and local**	**Study design**	**Device**	**Inclusion criteria**	**N**	**Birth GA** (wk)**, Birth weight** (g)**, Days old**^a^	**Initial ventilatory parameters**^b^	**Ventilatory targets**	**HFOV+VT outcomes compared to HFOV**
**Iscan et al.**^20^ Turkey	Randomized crossover trial: HFOV+VT or HFOV for 2 h with 15′ washout in AC+VT.	*VN500*	Inborn infants with 24–32 weeks, intubated in < 6 h of life due to RDS, with < 20% ET leak.	20	28.0 ± 2.4 1.080 ± 390 < 6 h of life	*F* = 10, I:*E* = 1:1, VThf = 2.0	pH 7.25–7.40; PCO_2_ 37.5–52.5	Lower hypocarbia and hypercarbia incidence and maintained VThf within the target range more consistently.
**Enomoto** **et al.**^21^ Japan	Crossover pilot: HFOV+VT for 6 h, then only HFOV for 6 h, without washout period.	*VN500*	Birth weight < 1 kg under HFOV with VT after 28 days of life.	6	23.7 ± 2.8 506 ± 56 50 ± 14.5	*F* = 12, VThf = 1.5 - 2.3	NA	Lower SpO_2_ fluctuations and SpO_2_ rates < 80%. Minute volume and DCO_2_ fluctuations increased after VT removal.
**Chen et al.**^22^ Taiwan	Retrospective study: from 2012 to 2016 (HFOV); from 2016 to 2017 (HFOV+VT).	HFOV: *SLE 5000* HFOV+VT: *VN500*	Preterm infants with AHRF refractory to AC.^d^	52	28.4 ± 4.3 1184 ± 690 6.8 ± 7.2	*F* = 10 - 15, I:*E* = 1:1 (HFOV) and 1:2 (HFOV+VT), VThf = 2	pH 7.25–7.45; PCO_2_ 35–55	Reduction in the combined outcome of death or BPD, and in hypercarbia episodes.
**Tana et al.**^23^ Italy	Retrospective study from 2016 to 2017 (HFOV); and prospective study from 2017 to 2018 (HFOV+VT).	*VN500*	Inborn infants with 24–27 weeks, intubated at birth due to RDS, electively HFOV ventilated, after a recruitment maneuver, with < 20% ET leak.	22	25.5 ± 1.1 721 ± 115 < 6 h of life	*F* = 15, I:*E* = 1:2, VThf = 1.5 - 2.0 (HFOV) and 1.5 - 1.8 (HFOV+VT)	pH 7.30–7.45; PCO_2_ 45–55	Lower VThf and PCO_2_ fluctuations after surfactant administration, and lower hypocarbia incidence.
**Lin et al.**^24^ China	Retrospective analysis of data between 2020 and 2022 using HFOV or HFOV+VT.	*SLE 6000*	Preterms after satisfactory PDA ligation and hemodynamically stable, with AHRF refractory to AC,^e^ and < 30% ET leak.	41	29.2 ± 2.9 1305 ± 271 26.4 ± 8.9	*F* = 8–10, I:*E* = 1:1, VThf =2 (HFOV+VT)	PCO_2_ 35–55	Lower hypocarbia and hypercarbia incidence, and lower duration of invasive ventilation.
**Solís-Garcia** **et al.**^19^ Spain	Prospective cohort between 2012 and 2013 (HFOV), and after a bundle^c^ between 2013 and 2018 (HFOV+VT).	HFOV: *Babylog 8000* HFOV+VT: *VN500*	Preterm infants born < 32 weeks, intubated with < 72 h of life due RDS, who received HFOV as rescue therapy f.	119	25.8 ± 2.8 865 ± 288 < 72 h of life	*F* = 8–10 (HFOV) and 15–17 (HFOV+VT) d	PCO_2_ 40–55	Higher survival free of grades 2–3 BPD, and free of chronic pulmonary treatments or respiratory hospital admissions at two years of age.

a Values shown as *mean ± SD.*

b Frequencies (F) are in Hz, and VThf (targeted in HFOV, or set in HFOV+VT) is in ml/kg.

c The quality bundle consisted of HFOV+VT as early rescue, using minimal volumes by increasing frequencies to 15–20 Hz, and use of minimally invasive surfactant therapy.

d Defined as an oxygenation index > 15 with PaO_2_ 〈 50 or SpO_2_ < 80% or PCO_2_ 〉 60.

e Defined as a peak inspiratory pressure > 28 cm H_2_O, or VT > 8 ml/kg, or PCO_2_ > 60 with pH < 7.20, or diffuse atelectasis.

f Defined as a FiO_2_ > 50% or PCO_2_ > 55 or peak inspiratory pressure > 15 cmH_2_O. AC, assisted-controlled ventilation; AHRF, acute hypoxic respiratory failure; Babylog 8000, Babylog 8000 ventilator Dräger, Lübeck, Germany. DCO_2_, carbon dioxide diffusion coefficient; ET, endotracheal tube; F, frequency (Hz); GA, gestational age in weeks; I:E, inspiratory to expiratory ratio; N, number of patients in each study; NA, not available; PCO_2_, carbon dioxide partial pressure (mmHg); PaO_2_, oxygen partial pressure (mmHg); RDS, respiratory distress syndrome; SD, standard deviation; PDA, patent ductus arteriosus; SLE, SLE ventilator; SLE UK, Croyden, United Kingdom; VN500, Babylog VN 500 ventilator Dräger, Lübeck, Germany; VT, tidal volume (ml/kg); VThf, high-frequency tidal volume (ml/kg).

**Table 2:** Studies ongoing and awaiting assessment.

**NCT05592431**. Effect of volume guarantee-high frequency oscillatory ventilation on cerebral blood flow in neonates. https://www.clinicaltrials.gov/study/NCT05592431.	
Status	Ongoing
Title	Effect of volume guarantee-high frequency oscillatory ventilation on cerebral blood flow in neonates
Methods	randomized clinical trial
Participants	Neonates with various causes of respiratory failure: respiratory distress syndrome (RDS), air leak syndromes, pneumonia, or pulmonary hemorrhage, failing with conventional ventilation (i.e., when conventional ventilation failed to maintain either oxygenation or ventilation) and are switched to HFOV as a rescue therapy.
Interventions	HFOV with VG (SLE6000;SLE) versus HFOV (SLE6000;SLE)
Primary outcome	Doppler cerebral blood flow velocity measurements
Starting date	2022

**ChiCTR2100052839.** A randomized controlled trial: invasive high-frequency oscillatory ventilation vs. high-frequency oscillatory ventilation combined with volume-guarantee for neonatal RDS. http://www.chictr.org.cn/showproj.aspx?proj=135584	
Status	Ongoing
Title	A randomized controlled trial: invasive high-frequency oscillatory ventilation vs. high-frequency oscillatory ventilation combined with volume-guarantee for neonatal RDS
Methods	Randomized clinical trial
Participants	1. Newborns diagnosed with respiratory distress syndrome within 12 h after birth and requiring tracheal intubation for high-frequency oscillatory ventilator treatment; 2. Gestational age ≥ 28 weeks; 3. Patients who started receiving non-invasive ventilation within 12 h after birth but changed to invasive high-frequency oscillatory ventilation within 3 days after birth.
Interventions	HFOV with versus without VG
Primary outcome	Bronchopulmonary dysplasia; Death at 36 postmenstrual weeks;
Starting date	2021

**JPRN-UMIN000022156**. The effect of volume-targeted ventilation in very low birth weight infants ventilated with high frequency oscillation: a randomized crossover trial. https://center6.umin.ac.jp/cgi-open-bin/ctr_e/ctr_view.cgi?recptno=R000025532	
Status	Awaiting assessment
Title	The effect of volume-targeted ventilation in very low birth weight infants ventilated with high frequency oscillation: a randomized crossover trial.
Methods	Randomized crossover trial
Inclusion criteria	(1) Birth weight < 1500 g (2) ventilated with HFOV (Babylog VN500) (3) age 1 week after birth (4) No change of ventricular settings (MAP, amplitude, VT, frequency)
Exclusion criteria	(1) unstable lung condition (i.e. pneumonia, pneumothorax) (2) unstable volume-targeted ventilation mode caused by leak (3) a case that the attending physician consider inappropriate
Interventions	HFOV with versus without VG (*n* = 20)
Outcomes	Primary: the ratio of desaturation (SpO_2_ < 80%) Secondary: heart rate; transcutaneous CO_2_; cerebral and peripheral blood flow
Notes	Started in 2016 and finished in 2020, awaiting publication

Two of the six included studies were crossover trials,^20^^,^^21^ and the four newer studies were cohorts.^19^^,^^22^, ^23^, ^24^ No significant differences were observed between the two sample groups from each study in terms of birth gestational age, birth weight, or days of life in any of the cohorts. In all studies, the mean birth gestational age and weight were lower than 30 weeks and 1500 g, respectively.

The studies differ in both the age of the patients and the conditions under which they were analyzed. Two studies evaluated infants who were intubated immediately after birth.^20^^,^^23^ The other four studies examined preterm infants who experienced failure of conventional mechanical ventilation: one analyzed patients within the first 72 h of life,^19^ another included infants with a mean age of 7 days,^22^ third studied patients with a mean age of 26 days who developed respiratory failure following patent ductus arteriosus ligation surgery,^24^ and the fourth focused on infants after 28 days of life.^21^

Definitions for failure in conventional ventilation differed across the four observational studies and were not provided in the crossover study. Only two studies provided complete data on exposure to antenatal steroids (nearly 45% received).^19^^,^^23^ Also, only two studies reported surfactant administration (all patients received).^20^^,^^23^

Each study adopted distinct initial ventilatory parameters. In three studies, different ventilatory parameters were also adopted for HFOV and HFOV with VT groups. The frequencies applied ranged from 8 to 17 Hz, and the desired (in HFOV) or adjusted (in HFOV with VT) VThf varied between 1.5 and 2.3 ml/kg. Moreover, frequency, amplitude, or VThf were subsequently adjusted to achieve slightly different pH and/or PCO_2_ targets, which ranged from 7.25 to 7.45 and 37.5 to 55 mmHg, respectively.

A meta-analysis of the outcomes could not be performed due to the differences in study design and incomplete reporting. The main findings from the included studies are:

• Two studies evaluated VThf variability, in preterm infants immediately after birth, and in both HFOV+VT was associated with lower rates.^20^^,^^23^ These two studies also evaluated amplitude variability and found no difference between ventilatory modes. The non-randomized crossover found an increase in minute volume fluctuations after VT removal, without difference in amplitude mean or variance.^21^
• Four studies evaluated hypocarbia episodes, and three found an association between HFOV+VT and lower rates.^20^^,^^22^, ^23^, ^24^ One study found a lower standard deviation of PCO_2_ with HFOV+VT.^23^
• Four studies evaluated hypercarbia episodes, and three found an association between HFOV+VT and lower rates.^20^^,^^22^, ^23^, ^24^
• Hypoxemia was the primary outcome of the non-randomized crossover study, which found a reduction in the percentage of time with SpO_2_ < 80% with HFOV+VT. (21). Two cohorts also evaluated hypoxemia incidence but did not find significant differences between groups.^22^^,^^24^
• Three cohorts evaluated the duration of mechanical ventilation; two found no significant difference.^19^^,^^22^ In the cohort that reported a significantly lower duration with HFOV+VT (3.7 ± 1.2 days versus 2.1 ± 1.0 days, *p* < 0.01), patients went to HFOV due to acute respiratory failure after patent ductus arteriosus ligation.^24^
• Three studies evaluated BPD rates at 36 weeks postmenstrual age (none as the primary outcome). Two reported no difference.^22^^,^^24^ One study reported a higher survival without BPD grades 2–3 in patients under HFOV with VT, but alongside other strategies compounding a quality improvement bundle.^19^
• None of the studies was designed to evaluate IVH or periventricular leukomalacia as primary outcomes. One study found no significant difference in IVH rates, evaluating preterm infants in the first weeks of life.^22^ Other study also found no difference in IVH or leukomalacia rates.^24^
• Three studies evaluated the mortality and found no statistical difference between groups.^19^^,^^22^^,^^24^ Of note, only one of the ongoing studies is randomized in design and aims to primarily assess clinical outcomes for this comparison.

### Quality assessment

The assessment of studies are summarized in Table 3. All studies reported no significant differences in baseline characteristics between the groups. One crossover was considered as having a high risk of bias due to the non-randomization process and the lack of a “washout” period.^21^ As for observational studies, the retrospective analysis, mainly in different periods, was a substantial trigger for biases. Two studies presented a critical risk of bias, mainly due to the measurement of the outcomes.^19^^,^^24^

**Table 3:** Risk-of-bias for randomized trials (ROB2) and the risk of bias in non-randomised studies of interventions (ROBINS-I).

**ROB 2 of crossovers**								
**Study**	Bias from randomization process	Bias arising from period and carryover effects		Bias due to deviations from intended interventions	Bias due to missing outcome data	Bias in measurement of the outcomes	Bias in selection of the reported result	**Overall risk of bias**
**Iscan et al.**^20^	Some concerns	Low		Some concerns	Low	Some concerns	Low	**Some concerns**
**Enomoto et al.**^21^	High	High		Some concerns	Low	Low	Some concerns	**High**

**ROBINS-I of retrospective**								
**Study**	Bias due to confounding	Bias in selection of participants into the study	Bias in classification of interventions	Bias due to deviations from intended interventions	Bias due to missing data	Bias in measurement of the outcomes	Bias in selection of the reported result	**Overall risk of bias**
**Chen et al.**^22^	Serious	Serious	Low	Serious	Moderate	Moderate	Moderate	**Serious**
**Tana et al.**^23^	Serious	Serious	Low	Serious	Moderate	Moderate	Moderate	**Serious**
**Lin et al.**^24^	Critical	Serious	Serious	Serious	Moderate	Critical	Serious	**Critical**
**Solís-Garcia et al.**^19^	Serious	Serious	Low	Serious	Moderate	Critical	Moderate	**Critical**

## Discussion

In this systematic review of six studies and 260 preterm infants, the authors compared HFOV with versus without VT regarding ventilatory and clinical outcomes. The report of included studies indicates that HFOV with VT, compared with HFOV, may reduce VThf variability, hypocarbia, and hypercarbia incidence.^20^^,^^22^, ^23^, ^24^ Findings on hypoxemia incidence and mechanical ventilation duration are mixed.^19^^,^^21^^,^^22^^,^^24^ Two studies found no difference in BPD rates, while one noted higher survival without BPD grades 2–3 under HFOV+VT.^19^^,^^22^^,^^24^ IVH, leukomalacia, and mortality outcomes were similar across groups.^19^^,^^22^^,^^24^

The proposal of VT on the HFOV is to reduce VThf fluctuations over time. In line with this, Belteki et al.^25^ reported a difference between set and delivered VThf of < 0.2mL/kg for 83% of the time when the mode was HFOV with VT. The two studies in this review reported lower VThf variability under HFOV with VT evaluated patients immediately after birth when there are rapid lung compliance changes.^20^^,^^23^ These studies, along with two additional ones, linked the use of HFOV with VT to lower rates of hypocarbia and hypercarbia – possibly a consequence of the reduction in VThf variability.^22^^,^^24^

It is unclear to what extent fluctuations in VThf would be associated with clinical outcomes, and the results presented in this review do not indicate a clear difference in the rates of BPD, brain injury, or mortality when comparing the two ventilatory modes. However, the lower hypocarbia and hypercarbia incidence with HFOV with VT presented in the included studies of this review, in preterm infants of different ages and under different clinical conditions, and the association between PCO_2_ variability and the increased risk of neurological injuries and mortality, is a condition that demands further investigation.^26^ In a recent animal study model under HFOV with and without VT, no differences were found in cerebral hemodynamics.^27^ A Of the trials ongoing (Table 2), two aim to evaluate the impact of VT on cerebral hemodynamics, and the third, BPD and mortality rates, allowing an evaluation of this hypothesis.

This systematic review has limitations. The authors were unable to perform a meta-analysis due to the differences in study design and incomplete reporting. Also, five studies were considered as having a relevant risk of bias. In three observational studies, the intervention group was assessed in a period after the control group, with bias related to advancements in neonatal care during the period. In addition, the equipment used was different across the studies and even between arms in the same study, raising concerns over measurement bias.^12^ In one study, outcomes were assessed before and after a quality improvement bundle that contained not only HFOV with VT (instead of HFOV applied) but also other strategies compounding a bundle of improvements.^19^ Moreover, the studies evaluated preterm infants at different ages, from birth to an average of 50 days of life, under different clinical conditions and with slightly different VThf and PCO_2_ target values, which may have contributed to the high heterogeneity found.

Despite the limitations above, the report of studies indicates that VT application in HFOV may reduce VThf variability, hypocarbia and hypercarbia, especially in extremely preterm infants in the first weeks of life. The current understanding that volutrauma is directly related to lung inflammation comes from studies that used conventional mechanical ventilation. Nonetheless, HFOV applies to very small tidal volumes; perhaps, in low volumes such as those applied in VAF, the effects of VT application are not so pronounced.

## Conclusion

The present results highlight the need for high-quality randomized prospective trials to compare HFOV with versus without VT, considering an optimal sample size to evaluate long-term outcomes such as BPD, leukomalacia, and mortality. The lower VThf variability reported when applying HFOV with VT may reduce clinicians’ willingness to randomize and may increase the dropout or crossover of patients under HFOV without VT who experience greater VThf and PCO_2_ fluctuations.

## Author contributions’ statement

Dr. Eduardo Orlandin formulated and structured the study, selected the included studies, collected data, performed the bias assessment and statistical analysis, drafted the preliminary manuscript, and critically reviewed and revised the manuscript.

Dr. Thaís Iwashita-Lages selected the included studies, collected data, performed the statistical analysis, coordinated and supervised data collection, drafted the initial manuscript, and critically reviewed and revised the manuscript.

Dr. Luis Oharomari performed the bias assessment, drafted the initial manuscript, and critically reviewed and revised the manuscript.

Milena Tomé, Dr. Mariana Zinher and Sofia Dias carried out the initial analyses, and critically reviewed and revised the manuscript.

Dr. Walusa Gonçalves-Ferri critically reviewed and revised the manuscript for important intellectual content.

All authors approved the final manuscript as submitted and agreed to be accountable for all aspects of the work.

## Funding

This study was conducted without funding support.

## Trial registration

Registered with the International Prospective Register of Systematic Reviews PROSPERO, under the assigned protocol CRD42023452788 on Aug 08, 2023.

## Ethical statement

This is a review study. The Research Ethics Committee of Clinics Hospital of Ribeirão Preto of the University of São Paulo has confirmed that no ethical approval is required.

## Conflicts of interest

The authors declare no conflicts of interest.

## Appendix Supplementary materials

Image, application 1
