# Supplementary material for: Volume-targeted on high-frequency oscillatory ventilation in preterm infants: a systematic review
Source: J Pediatr (Rio J). 2025 Mar 17;101(3):332–40. doi: 10.1016/j.jped.2025.01.012 (PMC12039505; doi:10.1016/j.jped.2025.01.012)
Supplement: Supplementary file 1 [file mmc1.docx]

**JPED-D-24-00582_ Supplementary material**

**Supplementary material**

Search strategy in all databases:

(neonatal OR neonate OR neonat* OR newborn OR premature OR preterm OR infant OR infants) AND (high-frequency OR “high frequency” OR “high frequency ventilation” OR “high frequency oscillatory ventilation”) AND (“volume guarantee” OR “volume-guarantee” OR “ volume targeted” OR ”volume-targeted”)

## Supplementary Table Excluded studies.

| **Study** | **Reason for exclusion** |
| --- | --- |
| Yau HCK, Cheung CSB, Yip SK, Lam HSHS. Volume-Targeted Versus Amplitude-Targeted High-Frequency Oscillatory Ventilation: A Retrospective Case-Control Study. Hong Kong Journal of Paediatrics 2022 27:1 (65-66). | Insufficient data (do not provide the number of subjects) |
| Jomily V, Manoj C. A Retrospective Pilot Study on the Effect of Volume Guarantee on High Frequency Oscillatory Ventilation in Neonates. Scholars Journal of Applied Medical Sciences, 2020. 08. 10-13. 10.36347/sjams.2020.v08i01.002. | Insufficient data (do not provide the mean gestational age and weight of subjects) |
| Enomoto M, Kikuchi S, Katayama Y, et alPO-0734 Effect Of Volume-targeted Ventilation In Extremely Low Birthweight Infants Under High-Frequency Oscillatory Ventilation. Archives of Disease in Childhood 2014;99:A495-A496. | Overlapped population with a included study (Enomoto et al. 2017 ^21^) |
| Iscan B, Duman N, Tuzun F, Kumral A, Ozkan H. Impact of Volume Guarantee on High-Frequency Oscillatory Ventilation in Preterm Infants: A Randomized Crossover Clinical Trial. Neonatology. 2015;108:277-82. doi: 10.1159/000437204. Epub 2015 Sep 1. PMID: 26330156. | Overlapped population with a included study (Iscan et al. 2015 ^20^) |
| Solís-García G, González-Pacheco N, Ramos-Navarro C, Rodríguez Sánchez de la Blanca A, Sánchez-Luna M. Target volume-guarantee in high-frequency oscillatory ventilation for preterm respiratory distress syndrome: Low volumes and high frequencies lead to adequate ventilation. Pediatr Pulmonol. 2021;56(8):2597-2603. doi:10.1002/ppul.25529 | Only HFOV-VG group without comparison |
| Tuzun F, Deliloglu B, Cengiz MM, Iscan B, Duman N, Ozkan H. Volume Guarantee High-Frequency Oscillatory Ventilation in Preterm Infants With RDS: Tidal Volume and DCO2 Levels for Optimal Ventilation Using Open-Lung Strategies. Front Pediatr. 2020;8:105. Published 2020 Mar 24. doi:10.3389/fped.2020.00105 | Only HFOV-VG group without comparison |
| Belteki G, Morley CJ. High-frequency oscillatory ventilation with volume guarantee: a single-centre experience. Arch Dis Child Fetal Neonatal Ed. 2019;104:F384-F389. doi:10.1136/archdischild-2018-315490 | Only HFOV-VG group without comparison |
| González-Pacheco N, Sánchez-Luna M, Ramos-Navarro C, Navarro-Patiño N, de la Blanca AR. Using very high frequencies with very low lung volumes during high-frequency oscillatory ventilation to protect the immature lung. A pilot study. J Perinatol. 2016;36:306-310. doi:10.1038/jp.2015.197 | Only HFOV-VG group without comparison |
| Zheng YR, Xie WP, Liu JF, et al. Impact of High-Frequency Oscillatory Ventilation Combined With Volume Guarantee on Lung Inflammatory Response in Infants With Acute Respiratory Distress Syndrome After Congenital Heart Surgery: A Randomized Controlled Trial. J Cardiothorac Vasc Anesth. 2022;36(8 Pt A):2368-2375. doi:10.1053/j.jvca.2021.10.012 | Population over 44 weeks of corrected gestational age, and after heart surgery |
| Ramos-Navarro C, González-Pacheco N, Rodríguez-Sánchez de la Blanca A, Sánchez-Luna M. Effect of a new respiratory care bundle on bronchopulmonary dysplasia in preterm neonates. Eur J Pediatr. 2020;179(12):1833-1842. doi:10.1007/s00431-020-03694-5 | Does not compare HFOV with versus without VG |

##

## 
